# Relapsing Classical Takotsubo Syndrome in a Postmenopausal Woman Successfully Managed with Psychology Consultations

**DOI:** 10.7759/cureus.5361

**Published:** 2019-08-10

**Authors:** Henrik L Hovgaard, Tomas Zaremba, Jens Aaroe

**Affiliations:** 1 Internal Medicine, Regions Hospitalet Randers, Aarhus C, DNK; 2 Cardiology, Aalborg University Hospital, Aalborg, DNK

**Keywords:** takotsubo cardiomyopathy, echocardiography, cardiac magnetic resonance imaging, cardio vascular events, cardiac imaging

## Abstract

Takotsubo syndrome (TTS) is an acute and often fully reversible heart failure condition. TTS was initially regarded as a benign syndrome, but it is known that TTS is associated with a mortality comparable to that of ST-elevation myocardial infarction. Interestingly, 2/3 of TTS occurrences are triggered by emotional or physical stressors. Meanwhile, the pathophysiology behind TTS is poorly understood. As no randomized trials exist to define the optimal treatment, current guidelines are based on expert opinion and the management of TTS-patients is often supportive.

We present the case of a postmenopausal woman with relapsing TTS from two different emotional stressors where the treatment was carried out in cooperation between psychiatric and cardiology specialists.

This case bears significance as severe relapsing TTS was managed successfully in collaboration between cardiologists and psychiatrists.

## Introduction

Takotsubo syndrome (TTS) is an acute heart failure syndrome most prevalent (90%) in postmenopausal women [1]. In 2/3 of cases, TTS is triggered by either severe physical or emotional stress, and in the remaining 1/3 patients, a plausible stressor cannot be found. TTS is characterized by transient hypokinesia and ballooning of the left ventricle, which often normalizes within 4-6 weeks [1].

In 1983 when our Japanese colleagues first described TTS, they thought the patients’ left ventriculogram resembled a traditional squid trap - a takotsubo. Although various English translations have been proposed (transient apical ballooning syndrome, left ventricular ballooning syndrome, broken heart syndrome), the original term is still used by the scientific community.

Clinically TTS is challenging to separate from an acute coronary syndrome (ACS), and patients often produce a slightly elevated troponin level leading to the diagnosis of myocardial infarction (MI) although coronary angiography (CAG) usually shows no significant obstructive lesions [2].

Retrospectively, it is now clear that 1-2% of all ST-elevation MI (STEMI) patients suffered from TTS rather than coronary obstruction [2]. Although TTS is most likely to be fully reversible, it should be considered a severe condition as 50% of patients experience cardiovascular complications such as ventricular or supraventricular arrhythmias, myocardial rupture or cardiogenic shock and the intrahospital mortality parallels that of STEMI (4-5%) [2].

Here we present the case of a postmenopausal woman seen in our clinic with relapsing classical TTS due to emotional stressors where the treatment was multidisciplinary between the cardiology and psychiatric departments.

## Case presentation

A 69-year-old caucasian woman was volunteering at the local soccer stadium when a spectator lost his temper and struck her. The physical trauma did not need emergency care, and she went home where she, for the first time, experienced worsening breathlessness and a tightening sensation around her chest. Five days later she was admitted with non-STEMI. Acute transthoracic echocardiogram (TTE) showed apical ballooning of the left ventricle and an ejection fraction (EF) of no more than 30% and an otherwise structurally normal heart. CAG revealed normal coronary arteries.

A few days later, she was discharged with a smaller dose of Bisoprolol (2.5 mg daily). Due to claustrophobia, our patient declined cardiac magnetic resonance imaging (MRI). One month later, TTE revealed completely normalized left ventricular function, and we performed no further follow up.

**Figure 1:**
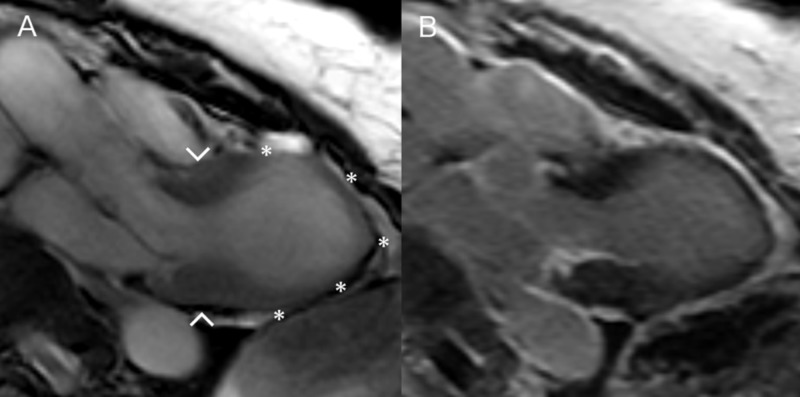
Cardiac MRI of our patient A: Systolic image with akinesia of the mid-ventricular and apical segments of the left ventricle (asterisks) with preserved contraction in the basal segments (arrowheads). B: Absence of late gadolinium enhancement.

Six months later, bad luck once again struck our now 70-year-old patient. Her former neighbor wandered off from his dementia nursery home to his previous address. He was picked up by the police. Later that night, our patient once again suffered transient chest pain. At admission to the emergency room, she was pain-free and presented an electrocardiogram (ECG) equivalent to STEMI and a troponin T of 1100 ng/L. Acute TTE revealed relapse of apical ballooning and an EF of 35%. CAG was once again without obstructive lesions; however, this time, our patient consented to cardiac MRI. The MRI confirmed the TTE findings of akinesia of the midventricular and apical segments of the left ventricle without necrosis or fibrosis as estimated by late gadolinium enhancement and an EF of 24% (Figure 1).

She was referred for psychiatric evaluation, and it was concluded that she did not fulfill the requirements for posttraumatic stress disorder but that she had an anxiety-driven personality structure. She was put on Citalopram and went through psychology rehabilitation with cognitive behavioral therapy and a TTE three months post-admission again showed completely normalized EF.

At the time of writing, she has had 18 uneventful months managed with Citalopram, psychology consultations and a small dose beta-blockers.

## Discussion

The pathophysiology of TTS is unclear. Animal studies suggest that catecholamine-induced myocardial stunning, multivessel coronary spasm, and microvascular dysfunction all contribute to a common phenotype [1]. Case reports from all over the globe testify to a heterogeneous composition of life events ranging from head trauma to alcohol withdrawal and Zumba that may trigger TTS [3-5].The diagnosis of TTS is often challenging. Several helpful diagnostic criteria have been proposed. The following list describes the latest diagnostic criteria from a consensus document in the European Heart Journal in 2018 [1].

1. Transient left ventricular dysfunction

2. An emotional, physical, or combined trigger may be present

3. Neurologic disorders, as well as pheochromocytoma, may trigger TTS

4. New ECG abnormalities may be present

5. Levels of cardiac biomarkers may be moderately elevated

6. Significant coronary artery disease is not contradictory of TTS

7. No evidence of infectious endocarditis may be present

8. Postmenopausal women are predominantly affected

No randomized, controlled trials exist to define the optimal treatment for TTS. Often, supportive treatment in the initial phase is preferred along with usual ACS treatment. Angiotensin Converting Enzyme (ACE)-inhibitors and angiotensin II receptor antagonists have retrospectively been shown to reduce one-year mortality along with the number of relapses (the recurrence rate is already low and estimated at 1.8% per patient-year) [1]. The use of beta-blockers is considered controversial [1,6]. Statins and aspirin are indicated only in cases where CAG reveals intracoronary plaques.

TTS patients should receive a cardiac MRI to determine the exact cause of myocardial injury and to exclude MI with non-obstructive coronary arteries or myocarditis and to evaluate the presence of an apical thrombus. As guidelines on the long-term management of TTS are lacking, follow-up regimens may vary between institutions. It does, however, seem reasonable to perform a follow-up TTE to exclude remaining signs of congestion and treat accordingly.

With this case report, we aim to draw attention to an interesting area within cardiology, which is currently subject to intense research and where current management rests on a weak scientific foundation.

## Conclusions

In this case of relapsing TTS, an emotional trigger was detected and treated accordingly with citalopram and psychology consultations. This example may inspire other cardiologists to look outside of our specialty in providing optimal treatment for our patients.
